# “*PPI? That sounds like Payment Protection Insurance*”: Reflections and learning from a substance use and homelessness study Experts by Experience group

**DOI:** 10.1186/s40900-021-00324-8

**Published:** 2021-11-20

**Authors:** Rebecca Foster, Hannah Carver, Jason Wallace, Alex Dunedin, Stan Burridge, Philip Foley, Bernie Pauly, Tessa Parkes

**Affiliations:** 1 Salvation Army Centre for Addiction Services and Research, Faculty of Social Sciences, 4S26 RG Bomont Building, University of Stirling, Stirling, FK9 4LA UK; 2 The Scottish Drugs Forum, 91 Mitchell Street, Glasgow, UK; 3 The Ragged University, Online Forum, Edinburgh, UK; 4 Expert Focus, Basildon, Essex UK; 5 Turning Point Scotland, 54 Govan Road, Glasgow, UK; 6 School of Nursing, University of Victoria, Victoria, Canada

**Keywords:** Patient and public involvement, Lived experience, Substance use, Homelessness

## Abstract

**Background:**

Patient and Public Involvement in research is important for citizenship, accountability and transparency, and has the practical benefit of helping to ensure its quality and applicability. Involving members of the public in research is becoming increasingly commonplace, in the UK and internationally. It is essential that public involvement is inclusive of individuals and their diverse life experiences, including challenging experiences that may be associated with stigma and social exclusion. The involvement of people with lived/living experience of substance use and homelessness in research is increasing in response to increased recognition of the importance of inclusion and the benefits conferred to research.

**Main body:**

In this commentary, we share our own experiences of being part of a Patient and Public Involvement group that was convened during a feasibility study of a peer-delivered harm reduction intervention. We are a diverse group but share experience of the field of substance use/homelessness, as people with lived/living experience, and as researchers and practitioners. We share our reflections and learning, as well as offer recommendations for researchers working in our field. Our group worked together to make a positive and deliberate contribution to the study. This did not happen by chance but required the development of mutual trust and respect, with each member having a commitment to support the group for its two-year duration.

**Short conclusion:**

It is important for researchers to appreciate that meaningful Patient and Public Involvement is very valuable but requires a commitment from all involved. Regarding our field of substance use and homelessness specifically, it is essential that people with these experiences have opportunities to contribute to research and can do so in a meaningful way. People with lived/living experience are able to bring to life the rich tapestry of others’ experiences. However, the involvement must be neither tokenistic nor indifferent to the wider challenges common to these experiences.

## Plain English Summary

This article describes the activities and lessons learned from the involvement of an Experts by Experience group in a National Institute for Health Research (NIHR) study. The study was about peer support for people experiencing homelessness and substance use challenges. The Experts by Experience group was made up of research team members, and a range of individuals from across the UK who had experienced homelessness and/or drug use and/or alcohol use, and who were recruited from a range of services who currently worked with people experiencing homelessness and/or substance use. The purpose of the group was to ‘sense check’ the study throughout. The group was a requirement from NIHR who funded the project. These groups are usually known as PPI groups (Patient and Public Involvement), but members of the group felt that ‘Experts by Experience’ was a more accurate term to describe the membership. The lived/living experience members’ knowledge of the topic, and experience of using the types of services and interventions involved in the study, helped to guide the study team. Study team members facilitated Experts by Experience group meetings, but everyone was equal. The group gelled and worked well together to oversee the study. This article describes the benefits of having Experts by Experience members as part of the project from the inception through to the end. Ensuring the group had autonomy enhanced the study and was a powerful and empowering experience for the lived/living experience members.

## Background

### Public and lived/living experience involvement

Public involvement is a requirement of research funded by the National Institute for Health Research (NIHR) in the United Kingdom (UK) [1]. Involving members of the public in research has the practical benefit of helping to ensure research quality and relevance. Additional reasons for inclusion include democratic principles relating to citizenship, accountability, and transparency [2]. NIHR’s express commitment to public involvement, established in 2016, signalled a cultural shift in the approach to conducting health research [3]. In the UK and internationally this has resulted in an expectation among funders, policy-makers and research organisations alike that Patient and Public Involvement (PPI) is part of research [4]. Indeed, involvement of the public in different stages of the research process, including in conception, design, conduct, and the dissemination of findings, has become an “expected norm” [5]. The recently published UK Standards for Public Involvement in Research [6] aim to provide a framework for good public involvement, and may help to prevent situations where PPI is exclusive or tokenistic rather than meaningful [7].

A critical, and pertinent, question concerns who is included as a ‘member of the public’. It is our view that involvement of members of the public should be sufficiently inclusive so that it is able to capture the diversity of individuals and the range of human experiences. Belle-Isle et al. (2016) observe (specifically in relation to those who use drugs, although it extends beyond this group) that including lived/living experience perspectives helps to ensure that research, as well as policies and services, are more relevant to the realities of the experiences of those affected [8]. Individuals with lived/living experience (also referred to as ‘peers’), are increasingly involved in research in the fields of substance use and homelessness. For example, they have been involved as general study advisors on formal groups, and provided input into the design, data collection and analysis of research [9]. They are also involved as researchers themselves – for instance, they are involved in conducting and analysing qualitative interviews [10]. There are now examples of new peer or lived experience researcher or research coordination roles within academic institutions where these roles bring significant value to whole research teams and programmes of work, rather than discreet projects. Individuals who have lived/living experience of substance use and homelessness are disproportionately affected by social exclusion and stigma [11–13]. The presence and impacts of (persistent) inequality and stigma bring into sharp focus the need for meaningful involvement in research. Finally, it is important to note that, while engaging with people who experience inequities is newer in all types of health research PPI, it has existed and is well-practiced in other fields, such as in Human Immunodeficiency Virus (HIV) and Community Based Participatory Research [14].

### SHARPS study

The Supporting Harm Reduction through Peer Support (SHARPS) study assessed the acceptability and feasibility of a peer-based relational intervention for individuals experiencing homelessness and substance use [15, 16]. Individuals experiencing substance use and homelessness in six sites in Scotland and England received practical and emotional support from a Peer Navigator for up to 12 months. These Peer Navigators had lived experience of homelessness and/or substance use. The study was undertaken between May 2018 and May 2020 and was led by TP. While PPI was required as part of the study by the funders, NIHR, SHARPS was rooted in a firm belief in these principles and of the benefits of involving those with first-hand experience of challenges in research. We embedded ‘lived/living experience’ in the study in several ways: through the study team with JW having lived experience; through the input of those with lived/living experience in the design and development of the intervention; through the delivery of the intervention by Peer Navigators; through the involvement of individuals with lived experience on the Study Steering Group (SSG) (JW and SB); through a specific PPI group; and through the involvement of peer researchers from the Scottish Drugs Forum who conducted qualitative interviews with intervention participants [15, 16]. The study PPI group is the focus of this commentary, but we wanted to share information about these other forms of lived/living experience inclusion in the study, to contextualise the PPI group and its role.

### Who we are and the focus of this commentary

Through the PPI group, the study team sought to include the voices, views and experiences of those members of the public with lived/living experience of homelessness and/or substance use who were living in Scotland and England. The remit of the group was to guide the study, check materials were suitable for participants, and ensure findings and dissemination activities were appropriate for the client group. Some of these activities are detailed below but included co-creating the initial intervention, making changes to the participant information sheets, supporting qualitative data analysis, and taking a lead on elements of study dissemination, for example through collectively writing this paper.

In this commentary, some members of this group and the study team share their reflections on their experiences, from inception through to closure of the group, which coincided with the duration of the study. We have written this commentary collaboratively, informed by publication and authorship recommendations provided by Richards et al. [17]. The group was chaired by JW and supported by HC and RF. As mentioned, JW was a member of the study team with lived experience of substance use and is a practitioner in the field. HC and RF were part of the study team and they worked with JW throughout and provided leadership, practical and administrative support to the group. AD, SB and PF have lived/living experience of some of the challenges detailed. TP led the study and had overall responsibility for the group. BP is a researcher and a practitioner in the field with extensive experience in community participatory research and a member of the study team. Although not involved in the group specifically, BP offered guidance and expertise on involving those with lived/living experience in the SHARPS study as a whole.

In this commentary, we reflect on how the group was meaningfully involved in the study and contributed to it, and how we collectively managed to achieve such involvement. We do this by reflecting on practical elements such as setting up the group, reimbursement, and communication. We hope that, in sharing this level of detail, other researchers, practitioners, members of the public, and individuals with lived/living experience, can benefit from our learning in this process. As Staley (2015) argues, detailed accounts of PPI can provide a rich source of learning [18]. Finally, we hope to reiterate the importance and value of involving members of the public who have experienced homelessness and/or substance use, in research.

### Getting started

Prior to the study starting, the team invited individuals with lived/living experience of homelessness and/or substance use to be involved in the group, through third sector organisations (with staff suggesting suitable individuals, which may have affected who was involved in the group and who was not given the opportunity). Some individuals had lived/living experience of substance use, some had experience of homelessness, while others had experience of both, alongside other, often related, challenges such as poor mental health. All individuals’ experiences and approaches to their recovery from substance use and homelessness were unique, and individuals were at different stages of their journeys. All those who expressed an interest in being involved in the group were able to join. The research team carefully planned the process in advance to ensure individuals were able to engage with the process, being attentive to issues of power and inequality. In this context it was crucial that the research team were psychologically/trauma informed (this was an important thread in the study intervention itself). This meant considering and being receptive to individual’s needs, behaviours and communication styles. The research team knew the importance of being flexible, consistent and delivering on promises and were highly committed to ensuring a good level of support was provided. Specific attention was paid to ensuring that all group members felt valued and listened to. One member reflected on how they felt at the start of the process and said “*I felt out of my depth… I just kept thinking ‘what can I offer those people?’. Thankfully the worry was for nothing as I was made to feel as important, we all did, as the Professors in the room*.”, highlighting that everyone was made to feel like equals.

Another member with lived/living experience commented:

*I find the red lines/no go areas of patient knowledge of science and medicine problematic. Through a lifetime of being spoken at by patronising and sometimes plainly ignorant doctors and institutions I had to understand the science and medicine then, when you get a grasp of the research, the protocols of the philosophy of science, and the language, one is found barred from the conversation. Until the privilege of such positioned people can enter into real dialogue with 'patients'/'lived experience'/individuals, discussing the sociology alone will not be sufficient. Those of us who have been forced to learn to survive have something to teach the vertical medicalised institution as much as we have something to learn from the stores of knowledge and experience they harbour. Similar history to that of the church really.*

The team recognised the contextual differences between and within the study sites in Scotland and England (for more details on the study please see [15, 16]). To take account of this it was hoped that the two PPI group members who lived in different parts of England could offer insight into these differences if/when needed. Despite attempts to include individuals from Black and Minority Ethnic/Global Majority and LGBTQ + communities, we were not able to recruit individuals with these identities/experiences. The resulting group comprised six individuals with lived/living experience (three men, three women), alongside JW, HC and RF. All group members were provided with a hard-copy detailed study information booklet, which also included details of planned meeting dates and payment. This acted as a form of ‘informal induction’ to help members understand their role, while being careful not to unduly formalise given the need to retain the authenticity of the lived/living experience [19] and the need for the research team to learn from *their* experiential knowledge. The intention was to have a female member as the Deputy Chair recognising that women tend not to put themselves forward for these roles: none of the female members volunteered/felt comfortable doing this so we agreed to go without one.

We had our first meeting in Glasgow, Scotland in May 2018 which was attended by authors AD, SB, PF, RF, HC and JW and the three other members of the group. After introductions to each other and to the study, the first thing we did was change the group’s name. No-one liked the term ‘PPI’, which, for several, had conjured associations with ‘Payment Protection Insurance’ (short-term income protection). One member suggested being called the ‘Experts by Experience’ (EbyE) group as an alternative, in recognition of the group’s expertise on account of their personal experiences and in keeping with other naming practices in the field. All agreed this was preferable. The team ensured they were consistent with this terminology in all subsequent communication, including referencing the group to others, using ‘PPI’ only where needed for clarity. At this first meeting, the group agreed ‘ground rules’ for how the group and meetings would operate. These rules were generally concerned with ensuring all members felt comfortable, welcome and respected. These rules were not prescriptive but encouraged collaborative working, respect and allowed members to be themselves and express themselves how they wanted. This is particularly important when involving those with experience of stigma and marginalisation who may have had negative experiences when interacting with institutions and processes, and where a formally established group by a university based research team may evoke unwelcome memories of these experiences. We also agreed on respectful communication and using non-stigmatising and inclusive language relevant to substance use, for example, avoiding the term ‘addict’ as some individuals experience this as stigmatising, dehumanising, and disempowering [20]. The appropriate use of language is important in all contexts but is particularly important in relation to substance use, as it has the power to shape perceptions and, in turn, minimise or entrench stigma [21]. It was important to revisit these ‘ground rules’ and use of language throughout the process, for example if there were disagreements.

### Meetings and communication

During the course of the two-year study, the EbyE group met face-to-face four times, and by conference call twice. Each meeting was focused on a particular aspect of the study which research team members (HC, RF and JW) set to correspond with study progress. All meetings allowed time for general conversation, checking in, and study updates and questions. This meant that they were flexible and each member was able to shape the direction of the meeting to an extent. The face-to-face meetings were in Glasgow, were four hours long, and included lunch and comfort breaks. Lunch and refreshments were provided. Conference calls were scheduled for 1.5 h.

RF/HC/an administrative colleague from the University of Stirling took detailed notes at all meetings and these were reviewed by JW before being sent round the group. The rest of the group was then invited to flag any inaccuracies or omissions at that stage. We discussed communication and which mediums people preferred, for example, email, WhatsApp. We decided to create a WhatsApp group to aid communication about meetings. One member did not participate in the WhatsApp group as they were concerned about the wider equalities and data protection implications of WhatsApp and similar platforms [22]. HC and RF communicated through the WhatsApp group as well as via email to ensure that everyone received information about meetings or other updates. While the WhatsApp group was mainly used to communicate information about the meetings, sometimes members would initiate other conversations, as a way of catching up, or to share a news article of interest. Having multiple methods of communication also ensured that members of the group had several sources of support: they could speak to members of the research team, with JW as the meeting chair, and with each other. We constantly reviewed the meeting and communication processes to ensure they were suitable for people’s needs, for example, allowing people to provide feedback via email instead of joining a teleconference meeting if they were more comfortable with this.

### Reimbursement

Everyone in the group was paid for their involvement, in line with INVOLVE guidance [23]. Members of the study team were involved in the group as part of their paid jobs, and the other members of the group were paid by the study team for their involvement; participation had been fully costed when the study was developed [23]. If meetings required preparation (for example, reading and reviews of material), preparation time was also reimbursed. The group could choose how they wanted to be paid: in cash/bank transfer, in ‘high street’ shopping vouchers, or in kind, for instance, one member chose to receive books. Payment delays were minimised and the research team prioritised ensuring timely processing of payments which can sometimes hamper PPI in large institutions. Over the course of the project, the university’s process for paying people for such activities changed and members were subsequently required to submit an invoice for each payment, which HC and RF supported. Cash payments were no longer allowed and payment had to be provided via bank transfer, although petty cash was allowed. To ensure members were paid quickly, invoices were submitted as soon as possible after the meetings. While we are (anecdotally) aware that there can be problems with paying people in cash for EbyE work, sometimes due to welfare benefits restrictions, in our experience it was reasonably straightforward and as members we were appreciative of the choice regarding these payments.

Members received travel expenses for attending meetings including public transport or taxi fares and were reimbursed at meetings (in cash) to ensure no-one was out of pocket. Accommodation costs were covered for the England-based group members, and meals and other incidental expenses were reimbursed (in cash) at meetings. RF and HC arranged all travel/accommodation with members to suit preferences.

### The contribution of the EbyE group to the SHARPS study

As mentioned, each meeting had a focus. For example, in meeting 1, the group reviewed participant-facing materials including the Participant Information Sheets (PIS); in meeting 2, the group reviewed a draft of the intervention guide; in meeting 3 the group reviewed a topic guide for the qualitative interviews with a sample of the SHARPS intervention participants; and in meeting 5, the group reviewed a sample of interview transcripts to identify and discuss emerging themes. The EbyE group’s input significantly enriched the study. We could give many examples of this but share five to give some insight, across the whole project.

Example One: part of the intervention development included creating a ‘guide’ or manual which the Peer Navigators would use to guide their work with participants. Firstly, members of the EbyE group attended the initial intervention development group to directly input into the creation of the intervention core components. Secondly, an early draft of the guide was presented and discussed at a subsequent EbyE meeting and changes were made in terms of: adding additional information about particular substances, nutrition, bullying, bereavement and parenting, among other things; providing a section on burnout for Peer Navigators; and creating a glossary of key terms and an index. These additions enhanced the amount of information the Peer Navigators had to hand, and ensured the guide was user-friendly.

Example Two: in the draft version of the PIS that intervention participants would receive to inform them of the study and what involvement would entail, we provided the name and contact details of the study ‘Chief Investigator’ (TP) as is standard practice. However, the EbyE group expressed that this term elicited unpleasant memories of interactions with the police service, or with those assessing their applications for welfare/benefits assistance. Given the client group, the study team members anticipated that many of the intervention participants would have experience of the criminal justice system [11]. The study team members were concerned that there was a risk of deterring those who may have been interested in taking part in the study and benefitting from doing so, or worse, triggering painful flashbacks, simply by using a term we had considered to be innocuous and regularly use within research. In response to this concern, across our participant materials, the team changed the terminology from ‘Chief Investigator’ to ‘Study Lead’. This has had a lasting impact as some of the researcher authors continue to use this alternative terminology in our other research projects, whenever possible. Another change suggested by members was to include photographs of the Peer Navigators on this PIS, so that participants could easily identify who these individuals were. Small changes were also made to other PIS and a consent form. Relatedly, changes were also made to the participant interview schedule, to ensure the wording of complex questions was appropriate.

Example Three: group members were also involved in qualitative data analysis. During one of the meetings, time was spent reading interview transcripts and discussing the key issues that arose. Members reflected on the data and their own experiences and these ideas informed the analysis and write up: for example, around the importance of peer support and lived experience. Group members provided insight into areas that academic researchers had not thought of, or placed high importance on. Members also raised questions around the use of prompts during the interviews and this was subsequently addressed by asking the peer researchers to use more prompts during follow up interviews.

Example Four: the group expressed concern that participants would be likely to feel ‘dropped’ or abandoned at the end of the 12 months when the intervention concluded. The study team had already considered this and were committed to sensitive, person-centred intervention ‘endings’, and participants also knew this when they agreed to take part. However, it was very important that this was managed sensitively, particularly considering the difficult life events many had experienced. The EbyE group’s heightened concern for the well-being of participants ensured the team paid additional and continued attention to this. For instance, the team spent more time supporting the Peer Navigators to support their participants as the intervention came to a close than originally intended. The team also asked the Peer Navigators to develop detailed and person-specific debrief plans three months prior to the close of the intervention, which were reviewed on an on-going basis as needed.

Example Five: members were involved in identifying dissemination approaches. Several ideas centred on providing easily accessible summaries of the study to participants and service users, and these are in the process of being created. This included short briefings, videos, press releases and items in relevant newsletters. The writing of this paper also provided an opportunity for the group to reflect on our experiences as a whole and share these with the academic community. Finally, we have worked with members of the group to inform a funding application for the next stage in the trial, a randomised controlled trial. Members provided insight into key issues such as intervention length and choice of primary/secondary outcomes to measure. We hope that if funded, the EbyE group will be re-established, with some new members, and we can continue and consolidate our learning together.

### Reflections: how do you ‘do’ meaningful involvement?

Expressed in simple terms, the group required commitment and input from all members to make it ‘work’. Insightful and impactful contributions, such as those described above, are unlikely to be offered if those being asked to make them do not feel comfortable, included and valued. All members needed to be committed to the SHARPS EbyE group and to make time to prepare for and attend meetings, alongside their other commitments and personal and professional responsibilities, over a two-year period. This applied whether they were a study team member organising train travel, or a lived/living experience member asked to review an interview topic guide for sensitivity and relevance.

From the perspective of the academic study team members, facilitating an EbyE group that has meaningful involvement and impact requires commitment to take on the administration of this: it is another formal group to manage in often complex and time sensitive studies. It also requires a commitment to provide ongoing informal support to ensure all members feel valued, comfortable and welcome, as well as continuous evaluation. These key components have recently highlighted by others [4, 24] and underpin the UK Standards for Public Involvement in Research [6]. Time needs to be built into study timelines to ensure researchers are able to give due attention to this work so that it does not feel rushed or ‘bolted on’.

#### Group respect

The study team members (RF, HC, JW) were mindful of the potential power imbalances that could have emerged between the study team members, and the lived/living experience members [25], and aimed to be responsive to these. There was a need to acknowledge the significant problems of unequal power and historical privilege that could shape personal interactions within large institutions such as universities, as one group member with lived experience stated:

*The way which institutions and power structures are functioning hobbles both the genuine workers and the peer researchers. The future necessarily involves an evolution where—for those who have developed the knowledge and skills—they can be valued for their contributions in holistic ways. There is something analogous to Kimberlé Williams Crenshaw's useful notionalising of 'intersectionality' going on where people and knowledge is theoretically erased due to the siloing and enclosure of fields. Are institutions, funding structures, ethics approaches and social valuations fit for purpose?*

One important point here is the importance of valuing contributions holistically—not ‘shoehorning’ people into bureaucratic processes to fit funding or other institutional processes or requirements. The point about siloing of academic disciplines and related erasure of human experience is also tremendously insightful and relevant to thinking about ways of intercepting and overtly addressing power within institutional contexts like universities. In terms of the practices we used to try to attend to power dynamics and the potential for inequities, after each meeting, RF, HC, and JW made reflective notes reflecting on how the meeting had gone, and how the dynamics could have been improved, if at all. From their perspective, the meetings went well, and this was confirmed with listening closely to feedback from the group who shared, confidentially, that they felt comfortable and felt that they were able to share their views freely. Some work was needed during the group process to ensure that all members had an equal voice, but all agreed that we achieved this. Members of the group came with very different experiences, with some being more confident and vocal than others. Through respectful dialogue, the creation of trust and relationships, and careful discussion about the best way to manage these issues, we were able to manage these. One member noted their experience of often being patronised by professionals and stated that the research team and EbyE members had entered into ‘real dialogue’ through the project, rather than having superficial level or tokenistic conversations which helped put them at ease and facilitate engagement.

There was some anxiety about the first conference call, in September 2018. HC and RF tried to alleviate this by giving clear instructions in advance. One member found the calls to be challenging so opted to provide feedback via email instead. This member was reassured that this was no problem, and the option was provided to everyone: our fluid, flexible, responsive and informal approach facilitated this. There was consensus that the face-to-face meetings were preferable to conference calls. Unfortunately, due to budget, it was not possible to change the conference calls to face-to-face meetings. Face-to-face meetings felt more productive and comfortable for us all. Nonetheless, we also reflect, given the current context of the COVID-19 pandemic, that it is essential for researchers working in this field to ensure that everyone is supported to access different communication methods, including phone and online video-conferencing facilities, and feel equipped to use these confidently. ‘Practice runs’ in advance of the planned meetings could help ensure this.

#### Changing circumstances and emerging opportunities

During the course of two years, people’s circumstances and lives changed. Two group members secured employment during the study and were unable to continue being part of the group. We decided not to replace them as we did not want a new member joining who did not have all of the background knowledge of the study or risk affecting the positive dynamic that had been fostered. Indeed, it is widely-recognised that it takes time for trust to be developed and in turn, for individuals to feel comfortable with self-disclosure [25]; this was essential to facilitate learning from the experiential knowledge of the lived/living experience members.

One member of the group (SB) was a member of both the EbyE group and the SSG. He was supported to give his time to these groups as part of his work time by his previous employer and did not receive any reimbursement for his participation. SB became a freelance consultant for his own company in June 2019 and wanted to continue involvement in both the EbyE and SSG. As a study team we wanted to continue to benefit from SB’s expertise, so we reached an agreement with him on how he could be reimbursed for his time thereafter. Having a member on both of these groups (as well as JW) meant that they could bridge the gap between the SSG and the EbyE group. Furthermore, study team members were committed to offering other opportunities to the lived/living experience members, and to supporting them with their personal and professional development. For example, inviting them to attend relevant events and seminars, and asking them to input on the team’s draft academic outputs for other projects, for which they were reimbursed. Some members have been/are part of similar EbyE groups on other research projects. Such activities ensured our approach to PPI was holistic and not a tokenistic exercise. One member reflected that being part of the SHARPS EbyE group increased their confidence and had a positive effect on their own recovery:

*In the first meeting I didn't really say anything as a lot of it went over my head and I didn't want to embarrass myself. But with the support in the room I found myself really enjoying it and knew I was contributing. The confidence I got from that really helped me in my recovery as I found I was afraid to put myself out of my comfort zone and a realisation that not all people would judge me as I'd found before. It also helped so much with me committing to things as it went on for 18 months which when I first sat at the first meeting I said to myself I'm not going to make the end of this. I was also so proud doing it and took a lot of joy telling people I was involved in such an important study, most people in my community didn't believe me! I also learned a lot about how the wheels turn slowly when you’re trying to change something and not to get down on myself. Through the skills and support I received I'm now involved in a few other projects and I can reassure people who maybe felt like me that it does and will get better, but like I learned from SHARPS you've got to stick it out.*

#### Close of group and continuing involvement

At the final meeting, it was clear how much some members of the group valued being part of it. Some members vocalised they were sad the group was coming to an end, and this was partly attributed to the sense of ownership and teamwork that had emerged. They felt proud of the group and what had been achieved, and some described it as ‘an honour’. RF, HC, and JW requested to stay in touch with the members on an individual basis if they wished, and to offer opportunities to members as they arose. They continue to be in touch with most of these members which is reflected in writing this paper together a year after the study formally ended. The study team (RF, HC, JW, TP, and BP) reflect that greater consideration should have been given to managing the endings of this group, in the same way that endings were managed for intervention participants. AD, SB and PF welcome this acknowledgement while recognising the work that had been done to prepare everyone for the group to close.

## Conclusions: closing reflections

In this commentary, we wanted to share our reflections and learning from our EbyE group. Much went well but, equally, there was room for improvement. The group made positive and impactful contributions to the SHARPS study, but this did not happen by accident; it required a sustained commitment and took a considerable amount of time from each member. Indeed, others have reflected on the resources required to ensure meaningful involvement [4, 24]. We feel that our EbyE group demonstrated that true power sharing among lived, practitioner and academic experiences was both possible and beneficial. As one member reflected: “*It is good that people are starting to be understood as good sources of information on their own lives and experience*”.

To express this colloquially, this collective effort was very much ‘worth it’. As researchers/practitioners we work in the field of homelessness and substance use, and as lived/living experience members we have ‘lived it’ and are now on different journeys. For us, the most important impact of our EbyE group is that its success contributes to evidence that demonstrates that people with lived/living experience of these challenges have much to offer, including to research. Without such insights, our study may well have experienced a variety of problems regarding recruitment of participants or might not have reported as positive a set of findings given the input into the intervention itself right at the start of the study. It is apparent, from our experiences, that a well-run and well organised EbyE group resulted in members having a very positive experience. Reflecting on our experiences we have put together the following recommendations for others working in the field. These are also in-keeping with those offered by others involved in working with those with lived/living experience of substance use specifically [26, 27]:

- involve as diverse a group of individuals as possible, but keep the group small to ensure relationships can be developed and everyone can be heard;
- carefully plan the process in advance, to facilitate engagement and ensure people feel valued, respected and listened to. Considering people’s needs, behaviours and communication styles in a psychologically/trauma informed way is essential
- ensure that everyone has an equal voice and feels that they have the ability to fully contribute by asking for regular feedback;
- provide detailed information about roles, responsibilities, expectations and activities at the beginning of the process, and provide reminders when necessary;
- ensure funding is adequate to cover all costs of travel, venue hire, catering, reimbursements, additional meetings (for contingency), accommodation and any unforeseen expenses;
- ensure individuals are appropriately reimbursed and in a timely way for their time and provide a range of payment options for individuals, if possible;
- have clear activities for each meeting and provide detail in advance;
- provide opportunities for informal chat and relationship building before, during and after meetings.

While we have focused our recommendations for researchers involving people with lived/living experience in research as part of EbyE/PPI groups, it is important for these to be considered within a broader context, including the structural dynamics involved, particularly when researching and working with people who have experienced marginalisation.

## Data Availability

Not applicable.

## Abbreviations

EbyE: Expert by Experience
HIV: Human immunodeficiency virus
LGBTQ+: Lesbian, gay, bisexual, transgender, queer, +includes any individual who feels they do not fit into these categories including intersex and asexual individuals
NIHR: National Institute for Health Research
PPI: Patient and Public Involvement
SHARPS: Supporting Harm Reduction through Peer Support
SSG: Study Steering Group
UK: United Kingdom
