# Correction to: *“PPI? That sounds like Payment Protection Insurance”*: Reflections and learning from a substance use and homelessness study Experts by Experience group

**DOI:** 10.1186/s40900-022-00352-y

**Published:** 2022-05-03

**Authors:** Rebecca Foster, Hannah Carver, Jason Wallace, Alex Dunedin, Stan Burridge, Philip Foley, Bernie Pauly, Tessa Parkes

**Affiliations:** 1 Salvation Army Centre for Addiction Services and Research, Faculty of Social Sciences, 4S26 RG Bomont Building, University of Stirling, Stirling, FK9 4LA UK; 2 The Scottish Drugs Forum, 91 Mitchell Street, Glasgow, UK; 3 The Ragged University, Online Forum, Edinburgh, UK; 4 Expert Focus, Basildon, Essex UK; 5 Turning Point Scotland, 54 Govan Road, Glasgow, UK; 6 School of Nursing, University of Victoria, Victoria, Canada

## Correction to: Research Involvement and Engagement (2021) 7:82 10.1186/s40900-021-00324-8

Following publication of the original article [1], the authors reported errors in the Funding section. The revised Funding section is indicated hereafter and the changes have been highlighted in **bold typeface**.

The incorrect Funding section reads:

The Experts by Experience group was funded by a study which was funded in whole by the National Institute for Health Research, grant number HTA 16/153/14. For the purposes of open access, the authors have applied a CC BY public copyright licence to any Author Accepted Manuscript version arising from this submission.

The correct Funding section should read:

**This project was funded by the Health Technology Assessment Programme (project number 16/153/14) and is now published in full in the Health Technology Assessment online journal [volume 26, and issue number 14, February 2022) and available here** https://www.journalslibrary.nihr.ac.uk/hta/WVVL4786#/abstract**Further information on the project is available at:** https://www.journalslibrary.nihr.ac.uk/programmes/hta/1615314/#/

**This report presents independent research commissioned by the National Institute for Health Research (NIHR). The views and opinions expressed by authors in this publication are those of the authors and do not necessarily reflect those of the NHS, the NIHR, MRC, CCF, NETSCC, the HTA programme or the Department of Health.**

All the changes requested are implemented in this correction and the original article [1] has been corrected.

## References

[1] Foster R, Carver H, Wallace J (2021). “*PPI? That sounds like Payment Protection Insurance*”: Reflections and learning from a substance use and homelessness study Experts by Experience group. Res Involv Engagem.

